# Genome-Wide Identification of LCY Genes in *Capsicum annuum* Reveals *CaLCYB1* as a Key Regulator of Carotenoid Biosynthesis with Implications for Abiotic Stress Tolerance

**DOI:** 10.3390/plants15091283

**Published:** 2026-04-22

**Authors:** Haiyang Yu, Ziji Liu, Xi Li, Tingli Wang, Shucan Liu, Shuo Xu, Qiaoyun He, Xiai Yang, Zhimin Li, Shitao Sun, Xiushi Yang, Genggui Liu, Xinhong Guo, Yanchun Deng, Chunsheng Hou

**Affiliations:** 1 Institute of Bast Fiber Crops, Chinese Academy of Agricultural Sciences, Changsha 410205, China; 82101235021@caas.cn (H.Y.); 19936703521@163.com (T.W.); liushucan@caas.cn (S.L.); 82101221142@caas.cn (Q.H.); yangxiai@caas.cn (X.Y.); lizhimin@caas.cn (Z.L.); sunshitao@caas.cn (S.S.); yangxiushi@caas.cn (X.Y.); 2 Graduate School of the Chinese Academy of Agricultural Sciences, Beijing 100081, China; 3 Tropical Crops Genetic Resources Institute, Chinese Academy of Tropical Agricultural Sciences, Haikou 571101, China; liuziji1982@163.com; 4 Changsha Academy of Agricultural Sciences, Changsha 410026, China; lx443100@163.com; 5 State Key Laboratory of Vegetable Biobreeding, Beijing Vegetable Research Center, Beijing Academy of Agriculture and Forestry Sciences, Beijing 100097, China; 18852864338@163.com; 6 Changsha Huir Biological-Tech Co., Ltd., Changsha 410125, China; liugenggui@163.com; 7 College of Biology, Hunan University, Changsha 410125, China; gxh@hnu.edu.cn

**Keywords:** pepper, stress response, carotenoids, lycopene β-cyclase, antioxidant activity

## Abstract

Carotenoids contribute to photoprotection and abiotic stress adaptation in plants, and lycopene cyclases (LCYs) occupy a key branch point in carotenoid biosynthesis. However, the composition and stress-responsive divergence of LCY genes in pepper remain insufficiently characterized. In this study, we identified six CaLCY genes in *Capsicum annuum* and analyzed their structural features and expression patterns under drought and salt stress. *CaLCYB1* showed the strongest and most consistent induction under both drought and salt stresses and was positively correlated with carotenoid accumulation, whereas the other CaLCY members exhibited distinct or negligible expression patterns. Transient overexpression of *CaLCYB1* significantly increased β-carotene and total carotenoid contents by 117.6% and 45.1%, respectively, relative to the empty-vector control, and also augmented ABTS^•+^ radical scavenging activity as well as ascorbate peroxidase (APX) activity. Conversely, virus-induced gene silencing (VIGS) of CaLCYB1 led to marked reductions in all of these parameters. Correlation analysis, together with gain- and loss-of-function assays, supports an important role of *CaLCYB1* in carotenoid accumulation and β,β-branch-related antioxidant responses under stress. Yeast two-hybrid screening identified three potential interactors of CaLCYB1, namely CaUBQ, CaLHP1, and CaLARP6B. This study provides a family-level characterization of LCY genes in pepper and identifies CaLCYB1 as a major stress-responsive member that directs carotenoid flux and enhances antioxidant capacity under abiotic stress.

## 1. Introduction

Carotenoids are ubiquitous isoprenoid pigments that are indispensable for plant growth, development, and adaptation to environmental stresses [1]. In photosynthetic tissues, carotenoids function primarily in light-harvesting and photoprotection, where they dissipate excess light energy and quench reactive oxygen species (ROS) to prevent photooxidative damage [2]. Additionally, carotenoids serve as precursors for key phytohormones, such as abscisic acid (ABA) and strigolactones, which are integral to plant stress signaling [3,4]. Given the dual role of carotenoids as antioxidants and hormone precursors, enhancing carotenoid accumulation has been recognized as a viable strategy to improve plant tolerance to abiotic stresses, including drought and salinity [5].

Lycopene cyclases (LCYs) catalyze a critical branching point in the carotenoid pathway by converting linear lycopene into cyclic α- or β-carotene [6,7]. Two main classes exist: lycopene β-cyclases (LCYBs), which introduce β-rings to form β-carotene, and lycopene ε-cyclases (LCYEs), which act in combination with LCYBs to form α-carotene [8]. Within this enzyme group, LCYB is the primary gateway to the β,β-branch, governing the production of β-carotene and downstream xanthophylls that contribute to antioxidant capacity and ABA biosynthesis [9,10,11]. By partitioning flux between the β,β- and ε,β-branches, lycopene cyclases influence the size and composition of β-carotene pools and the supply of precursors for abscisic acid (ABA) and strigolactones, thereby affecting photoprotective and stress-signaling pathways associated with abiotic stress tolerance [12]. In *Capsicum,* capsanthin–capsorubin synthase (CCS), a β-cyclase-related enzyme, produces the red keto-carotenoids of ripe fruits and can show LCYB-like activity in heterologous assays, indicating partial functional overlap with LCYB [13].

Increasing evidence has shown that altering LCYB expression or activity can significantly affect carotenoid accumulation and enhance stress tolerance in plants [14]. LCYB regulates the biosynthesis of β-carotene and downstream xanthophylls, which serve as photoprotective pigments and precursors for abscisic acid. Consistent with this role, overexpression of LCYB in transgenic sweetpotato increases total carotenoids and ABA and enhances tolerance to drought and salt, whereas LCYB loss-of-function in cereals and other crops reduces carotenoids, impairs ABA biosynthesis, and elevates oxidative stress [15,16,17]. Furthermore, the transcriptional regulation of LCYB is increasingly linked to stress signaling [18]. In *Torreya grandis*, a WRKY transcription factor directly activated *TgLCYB1* under waterlogging stress, boosting antioxidant enzyme activities and alleviating ROS damage [19]. In addition to LCYB, manipulating LCYE can also generate beneficial stress phenotypes in certain contexts. For example, overexpression of a celery LCYE (AgLCY-ε) in *Arabidopsis* increased lutein and β-carotene and enhanced salt tolerance under high NaCl treatment [20]. Moreover, a tomato lycopene ε-cyclase allele was reported to improve drought tolerance while also increasing fruit carotenoid content, suggesting that LCYE manipulation may yield beneficial stress phenotypes in crop contexts [21].

In many horticultural species, *LCY* constitutes a small gene family [22]. For instance, *Arabidopsis thaliana* and *Oryza sativa* have a single LCYB gene, while carotenoid-rich species, such as *Solanum lycopersicum* and *Carica papaya*, possess two or more LCYB isoforms with distinct expression patterns [23,24]. In *Capsicum annuum*, lycopene cyclases are encoded by a multi-member gene family that includes both LCYB and LCYE genes [25]. Peppers (*Capsicum* spp.) are economically important vegetable crops cultivated worldwide, valued for their nutritious, vividly colored fruits [26]. Ripe fruits accumulate abundant carotenoids, including the red keto-carotenoids capsanthin and capsorubin, unique to *Capsicum*, which confer the characteristic red pigmentation and substantial antioxidant value [27,28]. This rich carotenoid palette reflects the dynamic regulation of carotenoid biosynthesis. However, pepper is relatively sensitive to environmental stresses. Drought and salt stress frequently constrain pepper growth, yield, and fruit quality. Drought stress, in particular, is detrimental during pepper flowering and fruit set, leading to reduced fruit set and smaller fruits [29,30]. In the context of climate change, increasingly severe droughts and soil salinization pose urgent challenges, necessitating improved stress resilience in pepper [31]. Emerging evidence suggests that carotenoid metabolism is involved in pepper’s stress responses [32]. For instance, pepper seedlings exposed to drought or salt show significantly elevated carotenoid levels accompanied by strong induction of carotenoid biosynthetic genes [33]. Recent data show that *CaLCYB1* silencing severely reduces cold tolerance in pepper—lowering carotenoid levels, weakening antioxidant enzyme activity, and damaging photosynthesis—while exogenous brassinosteroid application restores those traits [34]. Thus, the LCY gene family in *Capsicum annuum* likely contributes not only to drought/salt responses but also to brassinosteroid-mediated chilling tolerance, acting as a hub linking carotenoid metabolism with multiple abiotic stress pathways. However, a systematic characterization of the LCY gene family and a functional dissection of key members’ roles in stress responses are still lacking.

This study aimed to bridge this knowledge gap by characterizing the LCY gene family in pepper and elucidating the functional role of *CaLCYB1* in drought and salt stress tolerance. We identified all LCY family members in the pepper genome and analyzed their phylogenetic relationships, gene structures, conserved motifs, and promoter cis-elements. Expression profiling under drought and salt stress identified *CaLCYB1* as a highly responsive candidate. We then employed a combination of transient overexpression (TOE) and virus-induced gene silencing (VIGS) to delineate its function, demonstrating that *CaLCYB1* is essential for enhancing carotenoid accumulation and antioxidant capacity under stress. Furthermore, we investigated its correlation with other pathway genes and identified potential protein interactors using yeast two-hybrid assays. Our findings identify CaLCYB1 as a key stress-responsive regulator that may favor carotenoid partitioning toward the β,β-branch, thereby contributing to antioxidant defense and stress tolerance.

## 2. Results

### 2.1. LCY Gene Family in Pepper: Members and Characteristics

Using BLASTP (BLAST+ 2.9.0) and HMM searches, we identified six CaLCY genes in the *C. annuum* genome. These comprised two lycopene β-cyclases (*CaLCYB1* and *CaLCYB2*), three lycopene ε-cyclases (*CaLCYE1*, *CaLCYE2*, *CaLCYE3*), and one capsanthin synthase (*CaCCS1-*a specialized functional β-cyclase). All six CaLCY proteins contain the conserved N- and C-terminal lycopene cyclase domains, supporting their assignment to the LCY family. In addition, CCS has been described as an evolutionarily specialized LCY-related carotenoid cyclase and was reported to retain LCYB-like β-cyclase activity in assays. Their lengths span 217–556 amino acids, with molecular masses ~24.7–62.3 kDa and theoretical pIs 4.73–8.85; subcellular localization predictions indicated chloroplast targeting for *CaLCYB1/2* and *CaCCS1*, plasma membrane association for *CaLCYE1*, and cytosolic localization for *CaLCYE2/3*; due to sequence shortness, localization predictions for *CaLCYE2/3* are tentative (Table S1). However, domain analysis showed that *CaLCYE2* and *CaLCYE3* retain conserved lycopene cyclase domains, suggesting that they are unlikely to be pseudogenes but may represent truncated genes with potential functional roles.

**Figure 1:**
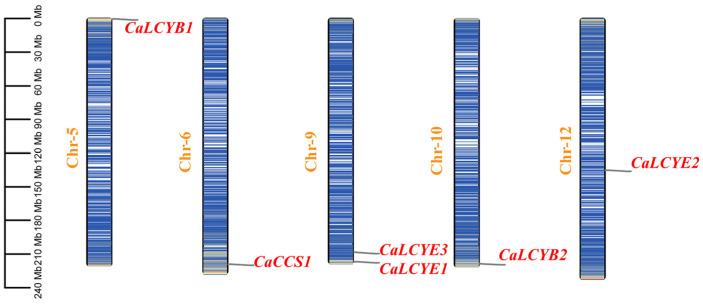
Chromosomal distribution of CaLCY genes. Chromosomes are to a common scale (0–240 Mb); six red-labeled loci (LCYB×2, LCYE×3, CCS×1) lie on Chr5/6/9/10/12, dispersed without tandem clusters.

Genomic mapping showed that the six CaLCY genes are dispersed on five chromosomes without clustering (Figure 1). Specifically, the two CaLCYB genes, three CaLCYE genes, and *CaCCS1* are located on chromosomes 5, 6, 9, 10, and 12. There is no evidence of recent tandem duplication events among them, implying that the gene family expansion (particularly the presence of multiple LCYE copies) likely resulted from older segmental duplications or whole-genome duplication in pepper’s lineage.

### 2.2. Phylogenetic Relationships of CaLCY Genes

**Figure 2:**
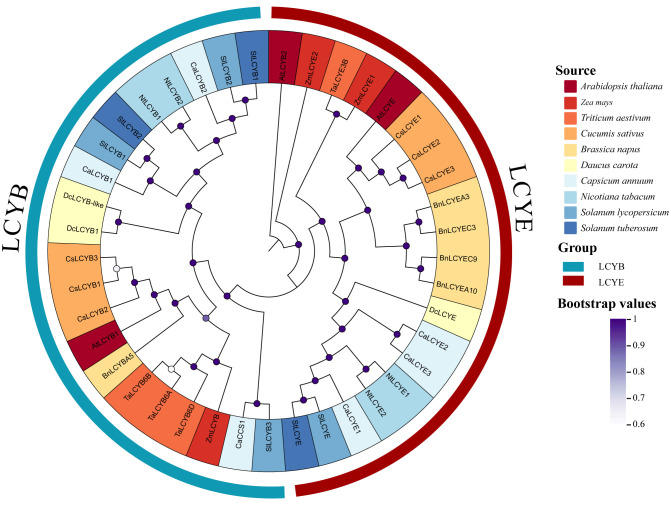
Neighbor-joining phylogeny of LCY proteins. A tree inferred from 41 LCY proteins across ten species (1000 bootstrap replicates) resolves two well-supported clades corresponding to LCYB and LCYE.

To elucidate the evolutionary relationships of pepper lycopene cyclases, we constructed a neighbor-joining phylogenetic tree with LCY proteins from pepper and other plant species. The analysis clearly separated the proteins into two major clades corresponding to β-cyclases (LCYB) and ε-cyclases (LCYE) (Figure 2). Consistent with their annotations, *CaLCYB1* and *CaLCYB2* clustered within the LCYB clade, while *CaLCYE1*, *CaLCYE2*, and *CaLCYE3* grouped within the LCYE clade. *CaCCS1*, a specialized β-cyclase functionally, was positioned adjacent to the core LCYB clade, indicating its evolutionary derivation from this group for capsanthin/capsorubin biosynthesis. Within the LCYB clade, *CaLCYB1* and *CaLCYB2* formed a distinct subgroup, suggesting a shared evolutionary origin from a gene duplication event specific to the *Solanaceae* lineage. This is analogous to the situation observed in tomato, where such duplication has led to functional divergence between paralogs. In the LCYE clade, the three pepper CaLCYE sequences were resolved as separate entities yet clustered closely with their Solanaceae homologs. The expansion to three LCYE copies in pepper—compared to the single copies typically found in tomato and Arabidopsis—implies potential neofunctionalization or subfunctionalization among these paralogs. These results suggest functional divergence between the LCYB and LCYE lineages and underscore the expanded and specialized nature of the lycopene cyclase gene family in pepper.

### 2.3. Conserved Motifs, Gene Architecture, and Promoter Cis-Elements of CaLCY Genes

**Figure 3:**
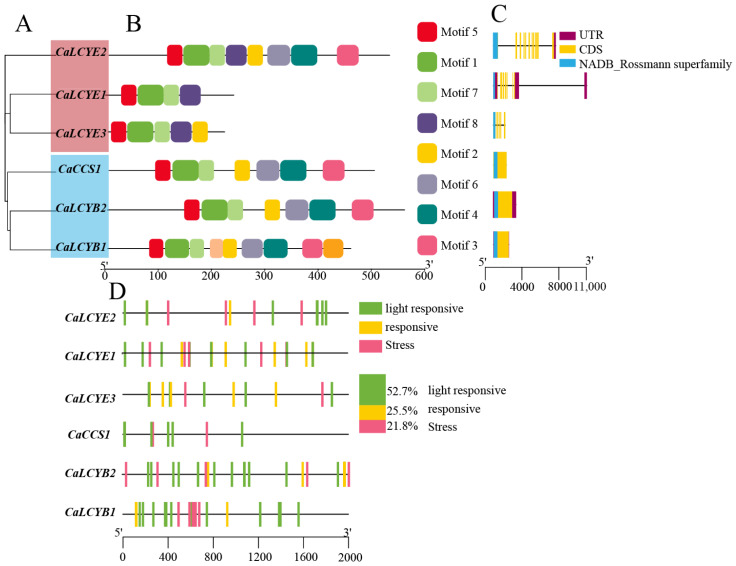
Phylogeny, conserved motifs, gene structures, and promoter cis-elements of the CaLCY family in pepper. (**A**) Neighbor-joining tree of CaLCY proteins, grouping LCYB and LCYE members. (**B**) Conserved-motif architecture predicted by MEME; colored boxes indicate motifs 1–8. (**C**) Exon–intron organization with UTRs (blue), CDSs (yellow), and annotated protein domains. (**D**) Cis-elements within 2 kb promoters; green, yellow, and pink marks denote light-, hormone-, and stress-responsive elements, respectively; proportions summarized at right (52.7%, 25.5%, 21.8%).

To further explore structural and regulatory features, we constructed a neighbor-joining tree using the six pepper CaLCY proteins (Figure 3A), which clearly separated LCYB and LCYE members, with *CaCCS1* clustering alongside the LCYB proteins. MEME analysis identified eight conserved sequence motifs that mapped to functional regions, including the N- and C-terminal lycopene cyclase domains and other catalytic signatures. Differences in motif composition and arrangement clearly distinguished LCYB from LCYE proteins; notably, *CaCCS1* exhibited an LCYB-like motif profile, consistent with its β-cyclase activity (Figure 3B). Gene structure analysis revealed that exon–intron organization mirrored these motif differences. *CaLCYB1* and *CaLCYB2* shared a broadly similar UTR/CDS organization, whereas the *CaLCYE* genes showed more variable gene structures and lengths.. In all genes, the mapped lycopene cyclase domains reside within coding exons. As shown in Figure 3C, the blue segments represented the conserved NADB_Rossmann superfamily domain, which was present in all six CaLCY proteins and further supports their classification within the LCY family.

To assess regulatory potential, we analyzed the 2 kb promoter regions upstream of each CaLCY gene. Light-responsive elements were the most abundant, accounting for approximately 52.7% of all predicted cis-elements, while hormone- and stress-responsive elements comprised about 25.5% and 21.8%, respectively. Notably, the *CaLCYB1* promoter contained a substantially higher density of stress-responsive elements compared to the other promoters (Figure 3D), consistent with its strong inducibility under drought and salinity stresses.

### 2.4. CaLCYB1 Is the Predominant Stress-Responsive LCY Gene Under Drought and Salt Stress

**Figure 4:**
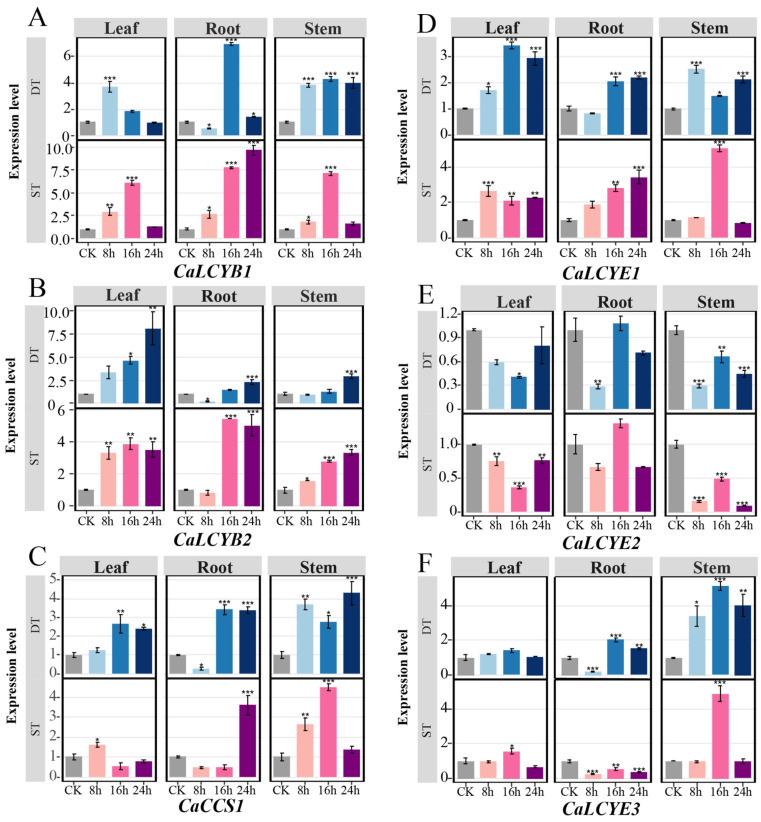
Expression dynamics of CaLCY genes under drought and salt stress. (**A**–**F**) Relative expression of *CaLCYB1*, *CaLCYB2*, *CaCCS1*, *CaLCYE1*, *CaLCYE2*, and *CaLCYE3* in leaf, root, and stem under drought (DS) and salt (SS) treatments at 0 h (CK), 8 h, 16 h, and 24 h. Bars show mean ± SE; asterisks denote significant differences relative to the corresponding control within the same tissue. Significance levels are defined as * *p* < 0.05; ** *p* < 0.01; *** *p* < 0.001.

To minimize developmental interference, we profiled CaLCY expression in roots, stems, and leaves of seedlings subjected to abiotic stress. To identify the most responsive genes, we performed qRT-PCR to analyze the expression of all six LCY genes in these tissues over a 0–24 h time course under salt and drought stress. Under salt stress, *CaLCYB1* showed the strongest induction, peaking in roots at 24 h (9.6-fold) , in stems (7.1-fold) and leaves at 16 h (6.1-fold). Under drought stress, its expression peaked earlier in leaves (8 h, 3.7-fold) and in roots at 16 h (6.9-fold) (Figure 4A). In contrast, *CaLCYB2* was primarily induced in leaves by drought at 24 h (8.1-fold), but remained largely unresponsive to salt stress. *CaCCS1* was strongly up-regulated by salt, particularly in roots (24 h, 3.8-fold) and stems (16 h, 4.3-fold), while drought caused only moderate increases. *CaLCYE1* was broadly up-regulated by drought, peaking in stems at 16 h (3.9-fold), with minimal salt response. Conversely, *CaLCYE2* was downregulated by both stresses across all tissues, with the lowest levels at 8–16 h. *CaLCYE3* expression increased mainly in stems, peaking at 16 h under both drought (4.8-fold) and salt (5.1-fold) stress (Figure 4B–F). Collectively, *CaLCYB1* exhibited the most consistent and pronounced upregulation under both stresses, supporting its designation as the predominant stress-responsive LCY gene in pepper.

### 2.5. Correlation-Based Screening Identifies CaLCYB1 as a Key Candidate Linked to Carotenoid Accumulation

**Figure 5:**
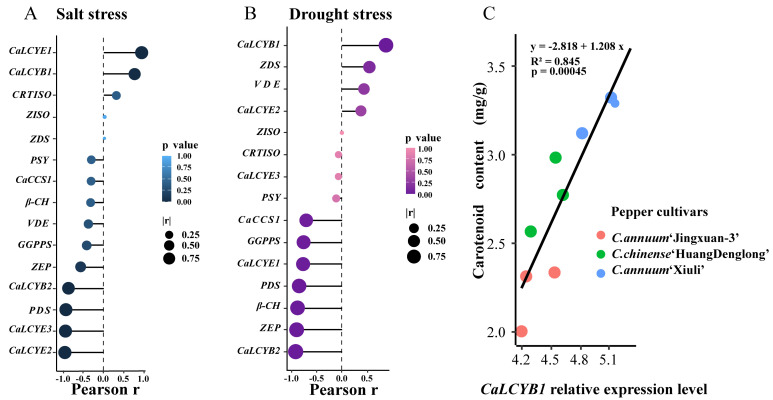
Correlation-based screening identified CaLCYB1 as a major positive correlate of carotenoid accumulation under drought and salt stress. (**A**) Pearson correlation analysis between carotenoid content and the expression of carotenoid-biosynthetic genes in roots under drought stress. (**B**) Pearson correlation analysis between carotenoid content and the expression of carotenoid-biosynthetic genes in roots under salt stress. (**C**) Correlation between carotenoid content and CaLCYB1 expression in fruits of three pepper cultivars under drought and salt stress, with the regression line, equation, coefficient of determination (R^2^), and *p*-value indicated.

To elucidate the relationship between the expression of LCY family members and carotenoid accumulation under stress conditions, we systematically evaluated candidate carotenoid-pathway genes by analyzing the correlation between transcript levels and carotenoid content under drought and salt stress. The results revealed that under salt stress, the expression level of *CaLCYE1* and *CaLCYB1* in seedling roots was strongly correlated with carotenoid content (correlation coefficient > 0.8), whereas under drought stress, the expression level of *CaLCYB1* and *ZDS* in roots showed a significantly positive correlation with carotenoid content (correlation coefficient > 0.4) (Figure 5A,B). Notably, *CaLCYB1* exhibited a stable and significant positive correlation with carotenoid accumulation under both stress conditions. This association was further validated in the fruits of three pepper types with distinct carotenoid profiles (*Capsicum annuum* ‘Xiuli’ (mature red), *Capsicum chinense* ‘HuangDenglong’ (yellow-fruited), and *Capsicum annuum* ‘Jingxuan-3’ (immature green)), where linear regression analysis demonstrated a strong positive relationship between *CaLCYB1* expression level and carotenoid content (R^2^ > 0.84, *p* < 0.01) (Figure 5C). Collectively, these findings highlighted *CaLCYB1* as a key candidate gene most closely associated with carotenoid accumulation during stress responses, providing a clear target for subsequent functional validation and mechanistic investigation.

### 2.6. CaLCYB1 Modulated Carotenoid Accumulation and Antioxidant Capacity

**Figure 6:**
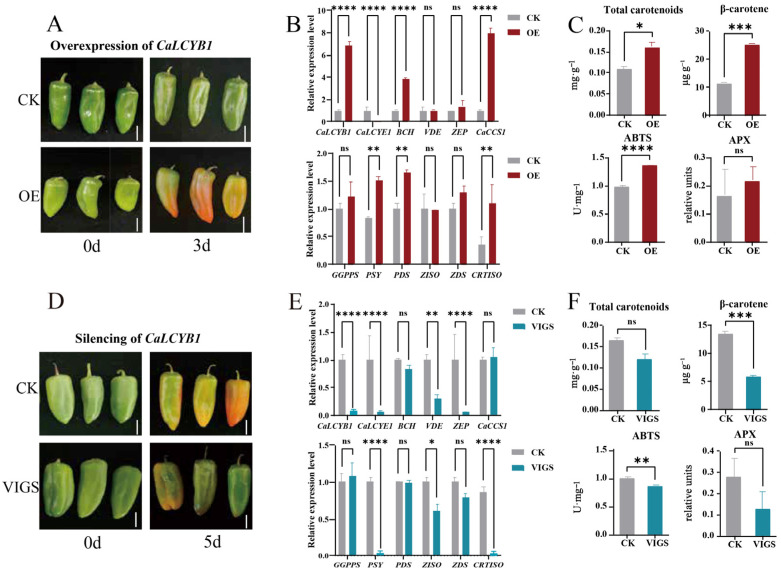
Effects of CaLCYB1 overexpression and silencing on pathway transcripts, carotenoid accumulation, and antioxidant indices. (**A**) Representative images of pepper fruit infiltrated for CaLCYB1 overexpression. (**B**) qRT-PCR analysis of CaLCYB1, β,β-branch genes (BCH, VDE, ZEP, CCS), and upstream biosynthetic genes (*GGPPS*, *PSY*, *PDS*, *ZISO*, *ZDS*, *CRTISO*) in CaLCYB1-overexpressing (OE) plants compared with the control (CK). (**C**) Total carotenoid content, β-carotene level, ABTS^•+^ scavenging activity, and APX activity in OE vs. CK. (**D**) Representative images of pepper fruit after CaLCYB1-silencing (VIGS) treatment. (**E**) Analysis of the same gene set in CaLCYB1-silenced (VIGS) plants. (**F**) Corresponding indices measured in VIGS plants. In panels (**B**,**C**,**E**,**F**,), data are presented as mean ± SE. Asterisks denote significant differences relative to the corresponding control. Significance levels are defined as ns, *p* ≥ 0.05; * *p* < 0.05; ** *p* < 0.01; *** *p* < 0.001; **** *p* < 0.0001.

To investigate the physiological function of *CaLCYB1*, we performed transient overexpression and VIGS-mediated silencing of this gene in pepper fruits and analyzed carotenoid accumulation, antioxidant capacity, and the expression of pathway-related genes. Three days after the overexpression treatment, the control fruits showed no obvious color change, whereas the infiltrated regions of 35S::CaLCYB1 fruits exhibited accelerated and intensified yellow–orange coloration (Figure 6A). Consistent with the phenotypic change, in fruits infiltrated with 35S::CaLCYB1, no significant differences were detected in the expression of upstream precursor-supplying genes, including *geranylgeranyl diphosphate synthase* (*GGPPS*), *phytoene synthase* (*PSY*), *phytoene desaturase (PDS*), *ζ-carotene isomerase* (*ZISO*), *ζ-carotene desaturase* (*ZDS*), and *carotenoid isomerase* (*CRTISO*) between CK and the treatment. In contrast, genes acting downstream of lycopene cyclization in the β,β-branch were preferentially upregulated: *CaLCYB1*, *BCH* (*β-carotene hydroxylase*) and *CaCCS1* (*capsanthin–capsorubin synthase*) were strongly induced, showing expression increases of more than 6-fold, 4-fold, and 7-fold, respectively (*p* < 0.01), although *ZEP* (*zeaxanthin epoxidase*) and *VDE* (*violaxanthin de-epoxidas*e) expression remained at baseline levels (Figure 6B). As a result, pigment levels were significantly elevated compared to empty-vector controls: β-carotene increased by 117.6%, and total carotenoids increased by 45.1% relative to the control (CK) (*p* < 0.05) (Figure 6C). Antioxidant capacity was also enhanced, with ABTS^•+^ scavenging activity rising by 36.5% (*p* < 0.01) and APX activity by 31.1% compared to CK (Figure 6C).

Five days after the silencing treatment, TRV2–CaLCYB1 fruits displayed delayed/attenuated coloration in the infiltrated tissues compared with CK (Figure 6D). In line with these changes, in TRV2–CaLCYB1-silenced plants, several carotenoid-pathway genes were downregulated. The transcript levels of *CaLCYB1*, *CaLCYE1*, *ZEP*, *CRTISO,* and *PSY* were markedly reduced, with transcript abundances in the control being approximately 12-, 15-, 16-, 20-, and 19-fold higher than those in the VIGS-treated samples, respectively. By contrast, *BCH* and *CaCCS1* showed only minor compensatory changes, whereas upstream biosynthetic genes (*GGPPS*, *PDS*, and *ZDS*) remained largely unaltered (Figure 6E). Correspondingly, pigment levels and antioxidant indicators shifted in the opposite direction: β-carotene decreased by 54.7%, total carotenoids by 27.1%, ABTS^•+^ scavenging by 12.9%, and APX activity by 53.7% relative to CK (Figure 6F). These results suggest that *CaLCYB1* redirects metabolic flux from lycopene into the β,β-branch, promoting the synthesis of xanthophylls and capsanthin, thereby expanding the pool of antioxidant pigments and enhancing ABTS radical scavenging and APX activity.

### 2.7. Subcellular Localization and Protein Interaction Network of CaLCYB1

**Figure 7:**
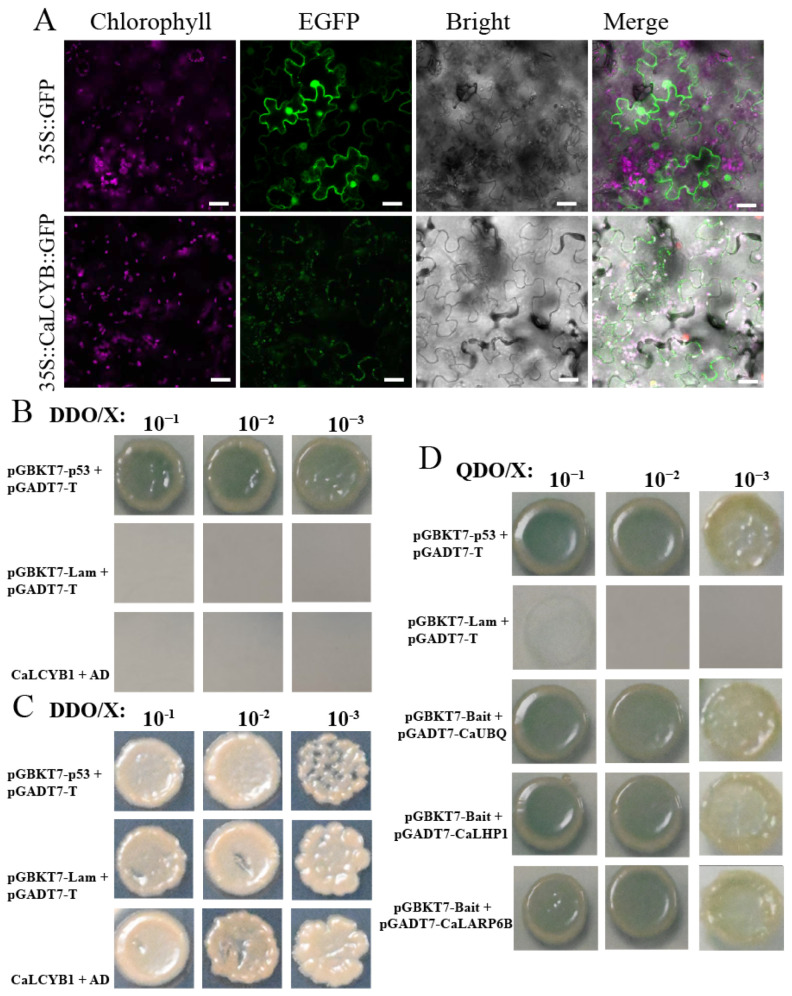
Subcellular localization and yeast two-hybrid analysis of CaLCYB1. (**A**) Subcellular localization of CaLCYB1 in tobacco epidermal cells. Scale Bar = 2 μm. (**B**) Auto-activation and control tests on QDO/X plates (SD/−Leu/−Trp/−His/−Ade + X-α-Gal): positive control (pGBKT7-p53/pGADT7-T) yields blue colonies; negative control (pGBKT7-Lam/pGADT7-T) shows no growth; BD–CaLCYB1 with empty AD shows no auto-activation. (**C**) Ten-fold serial dilutions (10^−1^, 10^−2^, 10^−3^) of the controls on QDO/X. (**D**) Pairwise assays of BD–CaLCYB1 (bait) with AD–CaUBQ, AD–CaLHP1, and AD–CaLARP6B spotted at 10^−1^, 10^−2^, and 10^−3^ on QDO/X. Colony growth with blue coloration indicates protein–protein interaction.

To further elucidate the functional mechanism of CaLCYB1, we investigated its subcellular localization and protein interaction network. Consistent with its predicted role in chloroplast-localized carotenoid biosynthesis, CaLCYB1 was confirmed to reside in the chloroplast (Figure 7A). We subsequently employed a yeast two-hybrid screen using BD-CaLCYB1 as bait to uncover potential regulatory partners. The bait protein showed no auto-activation (Figure 7B) and all control interactions—including the positive (pGBKT7-p53/pGADT7-T) and negative (pGBKT7-Lam/pGADT7-T) pairs—behaved as expected (Figure 7C). From the primary screening and subsequent pairwise validation, we identified three reproducible interactors: CaUBQ (→Ubiquitin), CaLHP1 (Like heterochromatin protein 1), and CaLARP6B (La-related protein 6B) (Figure 7D). Among them, CaUBQ and CaLHP1 showed strong interaction signals, sustaining yeast growth at 10^−3^ dilution on QDO/X/A plates, while CaLARP6B exhibited weaker but consistent growth up to 10^−1^–10^−2^ dilution. Functionally, CaUBQ encodes ubiquitin, the tag used by the ubiquitin–proteasome pathway for selective protein turnover; CaLHP1 is an H3K27me3-reading chromatin protein; and CaLARP6B is a La-related RNA-binding protein implicated in post-transcriptional control. Considered together, these interactors link CaLCYB1 to protein quality control, chromatin regulation, and RNA metabolism—processes commonly engaged during abiotic stress—thereby positioning CaLCYB1 within broader stress-response networks.

## 3. Discussion

In plants, carotenoid biosynthesis is tightly regulated, yet how metabolic flux is partitioned between the β,β- and ε,β-branches under stress remains incompletely understood [35]. Prior to this study, the functional roles of individual LCY genes in pepper stress adaptation were unclear, particularly given the expansion of the LCY family beyond the single LCYB typically found in model species. Previous research in pepper has largely emphasized pigment accumulation in fruits, leaving the genome-wide organization and stress-responsive roles of the LCY family unexplored [36]. Here, we systematically identified six LCY family genes in pepper, including two LCYB, three LCYE, and one CCS gene. This genomic framework not only clarifies the lineage-specific expansion of lycopene cyclases in pepper but also provides a basis for comparative analysis across species (Figure 1, Figure 2 and Figure 3). Our results suggested that CaLCYB1 was mainly associated with the β,β-branch under drought and salt stress (Figure 4, Figure 5, Figure 6 and Figure 7). In contrast, *CaLCYB2* showed much weaker stress responsiveness than *CaLCYB1*, suggesting divergence mainly at the level of stress-responsive regulation rather than demonstrated biochemical specialization.

In pepper, the lycopene cyclase gene family composition (two LCYB genes, three LCYE genes, and one CCS) is similar to that of other carotenoid-rich crop species but contrasts with the simpler complement in model plants, which typically carry only a single LCYB gene (Figure 1 and Figure 2) [37]. Notably, five of the six *CaLCY* genes are positioned near chromosomal ends (Figure 1). While the functional significance remains uncertain, telomeric regions are often associated with dynamic gene regulation in response to stress [38]. This may be compatible with the stress-responsive expression patterns of *CaLCY* genes. The two LCYB paralogs showed clearly differentiated transcriptional responses to abiotic stress, suggesting functional divergence between them. *CaLCYB1* functions as a positive stress-responsive regulator, promoting carotenoid accumulation in roots and fruits while being broadly upregulated across roots, stems, and leaves under drought and salt stress. Conversely, *CaLCYB2* is suppressed in roots, with its stress-responsive expression largely confined to stems and leaves (Figure 4 and Figure 5). Possible biochemical or structural differences between *CaLCYB1* and *CaLCYB2* also cannot be excluded, as lycopene cyclases are known to differ in catalytic specificity and in key residues that affect cyclization activity.

Notably, the consistent downregulation of *CaLCYE2* under both drought and salt stress may be compatible with reduced flux into the ε,β-branch, which could indirectly favor carotenoid partitioning toward the β,β-branch [39]. This organ- and context-specific deployment likely contributes to the partitioning of carotenoid metabolism between photosynthetic chloroplasts and fruit chromoplasts in pepper. In this study, we first performed stress-responsive expression profiling in seedling roots, stems, and leaves to identify LCY family members responsive to drought and salt stress. We then conducted functional validation in pepper fruits, which are rich in carotenoids and therefore well suited for transient overexpression and VIGS assays [40,41]. Accordingly, the fruit-based experiments were used to assess the role of CaLCYB1 in carotenoid accumulation and antioxidant-related responses. Nevertheless, its direct functional role in vegetative tissues under drought or salt stress remains to be determined.

Through functional assays, we established that *CaLCYB1* serves as a key regulatory node under abiotic stress conditions. Under drought stress, total carotenoid content increased concurrently with *CaLCYB1* expression, indicating a coordinated enhancement of carotenoid biosynthesis. Under salt stress, *CaLCYB1* expression correlated positively with *VDE* but negatively with *BCH* (Figure 5A,B), which may reflect a coordinated adjustment of downstream carotenoid metabolism that helps maintain the β-carotene pool under stress [42,43]. Fruit-based gain- and loss-of-function assays support a role of CaLCYB1 in regulating β,β-branch carotenoid accumulation and antioxidant-related responses. Transient overexpression of *CaLCYB1* increased β-carotene by approximately 117.6% and elevated total carotenoid levels, which was accompanied by enhanced ABTS^•+^ scavenging capacity and increased ascorbate peroxidase (APX) activity (Figure 6C), which was consistent with the previous study [44]. In contrast, silencing *CaLCYB1* led to a 54.7% reduction in β-carotene and a marked decline in antioxidant capacity. Transcript profiling revealed that downstream β,β-branch genes, including *BCH*, *ZEP*, and *CCS*, were upregulated during *CaLCYB1* overexpression, whereas upstream biosynthetic genes (e.g., *GGPPS*, *PSY*, *PDS*, *ZISO*, *ZDS*, and *CRTISO*) remained largely unaltered (Figure 6E). These results suggested that *CaLCYB1* mainly affected downstream carotenoid accumulation, while having little effect on the expression level of upstream pathway genes. Changes in downstream genes such as *BCH*, *ZEP*, and *CaCCS1* were also observed, supporting a role for CaLCYB1 in carotenoid regulation under stress.

Beyond transcriptional regulation, yeast two-hybrid screening revealed that CaLCYB1 interacts with proteins involved in ubiquitin-mediated turnover (CaUBQ), chromatin remodeling (CaLHP1), and RNA metabolism (CaLARP6B) (Figure 7). CaUBQ encodes ubiquitin, which functions in the ubiquitin-proteasome pathway for selective protein degradation [45,46], suggesting a potential role in regulating CaLCYB1 protein stability. CaLARP6B is a La-related RNA-binding protein involved in post-transcriptional regulation [47,48], raising the possibility of post-transcriptional control over CaLCYB1 via cytoplasmic mRNA metabolism before chloroplast import. CaLHP1 is a nuclear-localized chromatin protein that recognizes H3K27me3 [49,50], implying that it may indirectly affect CaLCYB1-related processes through nucleus-to-chloroplast communication rather than direct physical interaction. Taken together, these candidate interactors suggest that CaLCYB1 may be associated with protein quality control, chromatin-related regulation, and RNA metabolism, which are often involved in abiotic stress responses. However, these Y2H results should be regarded as preliminary, and the proposed interactions still require in planta validation. Future work should further examine their functional relevance in different tissues and under stress conditions. While our results suggest that CaLCYB1 contributes to enhanced antioxidant capacity, direct physiological evidence for whole-plant stress tolerance remains to be established. Future studies using stable transgenic plants, combined with comprehensive physiological assays (e.g., survival rate, electrolyte leakage, and MDA content) under drought and salt stress, would help to further validate this role.

## 4. Materials and Methods

### 4.1. Plant Materials and Stress Treatments

The sweet pepper cultivar ‘Fuxiang Xiuli Hongjiao’ (*Capsicum annuum* L.) was purchased from Hunan Xingshu Seed Co., Ltd. (Changsha, China), for all experiments. Seedlings were grown in a mixed substrate of nutrient soil, perlite, and organic fertilizer at a ratio of 5:3:2 (*v*/*v*/*v*), which provided a relatively loose, well-aerated, and well-drained growth medium. The plants were maintained in a controlled growth chamber at 25 °C and 60% relative humidity under a 16 h light/8 h dark photoperiod. At the four-leaf stage, uniform seedlings were subjected to drought or salt stress treatments. Drought stress was simulated by applying 20% (*w*/*v*) PEG-6000 to the root zone, and salt stress was imposed by irrigating with 200 mM NaCl solution. Untreated plants maintained under the same growth conditions served as controls. Root, stem, and leaf tissues were sampled at 0 h (pre-treatment), 8 h, 16 h, and 24 h after treatment. Three independent biological replicates were collected for each treatment and time point. All samples were immediately frozen in liquid nitrogen upon harvest and stored at −80 °C until subsequent analyses.

To investigate the variation in carotenoid content between *Capsicum chinense* and *C. annuum*, three cultivars with distinct fruit color phenotypes at harvest were selected: the yellow-fruited *C. chinense* ‘HuangDenglong’, and the two *C. annuum* cultivars ‘Jingxuan-3’ (immature green) and ‘Xiuli’ (mature red). All plants were cultivated under controlled greenhouse conditions at the National Bast Fiber Crops Germplasm Nursery of the Institute of Bast Fiber Crops, Chinese Academy of Agricultural Sciences (IBFC, CAAS) in Changsha, China (28°12′58″ N, 112°53′49″ E). The experiment was conducted using a completely randomized design with three treatment groups: control (CK), drought stress, and salt stress. Plants in the control group were irrigated regularly with deionized water to maintain optimal soil moisture. For experimental design and stress treatments, no combined stress treatment was included in this study. Uniform seedlings at the four-leaf stage were divided into three groups: control (CK), drought stress, and salt stress. Control plants were irrigated regularly with deionized water to maintain optimal soil moisture. Drought stress was imposed by completely withholding irrigation for 7 days, whereas salt stress was applied by irrigating the root zone with a 200 mM NaCl solution prepared in deionized water for 7 days.

### 4.2. Genome-Wide Identification of LCY Family Genes

To identify LCY family genes in pepper, we retrieved the *Capsicum annuum* reference genome (UCD10X v1.1) and its annotation from NCBI [51]. Candidate homologs were detected by BLASTP (BLAST+ 2.9.0) (e-value < 1 × 10^−10^) and by querying the Pfam lycopene-cyclase HMM (PF05834) with HMMER 3.4 (https://pfam.xfam.org/). In addition, all candidate sequences identified by BLASTP and HMMER were further examined for the presence of the canonical LCY domain (a Rossmann-fold NAD(P)-binding domain) using the SMART (version 10) and NCBI CDD (CDD, version 3.21) tools [52,53]. Candidates lacking the characteristic domain were discarded. Only sequences containing the complete NAD(P)-binding Rossmann-fold domain were retained as members of the pepper LCY gene family. Physicochemical properties of the identified LCY proteins, including amino acid length, molecular weight (Mw), theoretical isoelectric point (pI), instability index, aliphatic index, and grand average of hydropathicity (GRAVY), were calculated using the ExPASy ProtParam tool (https://web.expasy.org/protparam/; accessed on 20 August 2025). Subcellular localization predictions were performed using the online prediction website WoLF PSORT (https://wolfpsort.hgc.jp/; accessed on 20 August 2025) to infer the likely cellular compartment for each protein.

### 4.3. Phylogenetic Analysis and Chromosomal Mapping

The chromosomal positions of CaLCY genes were retrieved from the genome annotation (GFF) file. A map of gene locations was constructed with TBtools (v2.119), showing the distribution of CaLCYB, CaLCYE, and CaCCS genes on pepper chromosomes [54]. To investigate the evolutionary relationships of pepper LCY proteins with those of other species, we performed multiple sequence alignment and phylogenetic tree construction. LCY protein sequences from pepper and from nine other representative plant species were collected. Multiple-sequence alignment (ClustalW) and phylogenetic reconstruction (neighbor-joining, 1000 bootstrap replicates) were performed in MEGA 11, resolving LCYB and LCYE clades. The resulting phylogeny included a total of 41 LCY family sequences and distinguished the LCYB and LCYE clades. For visualization, the tree was annotated and rendered using the Chiplot platform [55]. A separate phylogenetic analysis focusing on the pepper CaLCY proteins was also performed: an NJ tree was constructed to observe clustering of CaLCY genes within pepper.

### 4.4. Gene Structure, Conserved Motifs and Promoter Cis-Elements

To characterize structural and regulatory features of the pepper CaLCY genes (Table S2), we analyzed protein motifs, gene architecture, and promoter cis-elements in the same workflow. Conserved motifs were identified using MEME (http://meme-suite.org/tools/meme/; accessed on 22 August 2025) with 8 motifs and the default settings. These motifs were then annotated against Pfam and NCBI CDD (https://www.ncbi.nlm.nih.gov/cdd/; accessed on 24 August 2025)to map the functional regions, including the canonical NAD(P)-binding Rossmann-fold. Gene structure (exon–intron organization) was determined by aligning coding sequences to their genomic loci and visualized using TBtools. We recorded the number and length of exons/introns, as well as 5′/3′ UTRs, and overlaid conserved domain positions on each gene model. For regulatory context, the 2 kb upstream region from the translation start site of each CaLCY gene was scanned with PlantCARE to catalog cis-elements. We emphasized light-responsive motifs, hormone-responsive motifs, and stress-responsive motifs. Element counts were normalized to promoter length and summarized by functional category across the family. Integrating motif/domain distribution with exon–intron architecture and promoter composition allowed us to infer putative regulatory modules and to prioritize CaLCY members—particularly those with stress-enriched promoters—for downstream functional assays.

### 4.5. Quantitative RT-PCR (qRT-PCR) Expression Analysis

Total RNA was extracted from pepper tissues (roots, stems, and leaves) from control and stressed plants using VeZol Reagent (R411, Vazyme, Nanjing, China). First-strand cDNA was synthesized from 1 µg total RNA per sample, and any residual genomic DNA was removed by using ABScript Neo RT Master Mix for qPCR with gDNA Remover (RK20433; ABclonal Biotechnology Co., Ltd., Wuhan, China). was performed using Gene-specific primers for each targeted gene were designed for qRT-PCR using the online NCBI Primer-BLAST tool (accessed on 20 July 2025) to amplify ~100–200 bp regions with high specificity primers for each target (Table S3). The pepper E2 gene was used as the internal reference gene for normalization because of its fundamental role in maintaining cellular proteostasis and its reported stable expression under abiotic stress conditions in pepper. A single peak was observed in the melting curve analysis. QRT-PCR was performed on a Bio-Rad CFX96 using a BrightCycle Universal SYBR Green qPCR Mix with UDG (RK21219; ABclonal Biotechnology Co., Ltd., Wuhan, China) according to the manufacturer’s instructions. Each biological sample was run in triplicate technical replicates, and no-template controls were included to check for contamination. Additionally, data were analyzed using the 2^–ΔΔCt^ method to determine relative gene expression, with stressed samples compared to the 0 h control group.

### 4.6. Transient Overexpression of CaLCYB1 in Pepper Fruits

We performed transient expression in pepper fruits because they accumulate high carotenoid levels and are readily infiltrated by Agrobacterium. The full-length *CaLCYB1* CDS was cloned into pBI121 under the CaMV 35S promoter (primers in Table S4). The resulting construct was introduced into Agrobacterium tumefaciens GV3101, and the empty vector served as the control. Agrobacterium cultures at OD600 ≈ 0.6 were harvested and resuspended in infiltration buffer (10 mM MgCl_2_, 10 mM MES, pH 5.6, 150 µM acetosyringone). Green-mature fruits (30–35 days post anthesis, DPA) were infiltrated using a needleless syringe (Gemtier Medical (Shanghai) Inc., Shanghai, China) through the pedicel site and maintained under normal conditions. Infiltrated tissues were collected 3 days post-infiltration; *CaLCYB1* overexpression was verified by qRT-PCR, and carotenoid content and antioxidant activity were quantified.

### 4.7. Virus-Induced Gene Silencing of CaLCYB1

Pepper fruits were used for virus-induced gene silencing (VIGS) because of their high carotenoid content and infiltration efficiency. A 250 bp *CaLCYB1*-specific fragment was selected using the SGN VIGS tool (https://vigs.solgenomics.net/; accessed 15 September 2025), amplified, and cloned into the TRV2 vector to generate pTRV2-CaLCYB1 (primers in Table S4). pTRV1 was used as the helper, and empty pTRV2 served as the control. The constructs were introduced into Agrobacterium tumefaciens GV3101. Cultures were grown to OD600 ≈ 0.8, resuspended in infiltration buffer, and infiltrated into green-mature fruits at a 1:1 ratio (pTRV1:pTRV2) using a needleless syringe (Gemtier Medical (Shanghai) Inc., Shanghai, China). After 24 h in darkness, fruits were returned to normal conditions. *CaLCYB1* silencing was confirmed by qRT-PCR at 5 days post-infiltration, and infiltrated tissues were used for carotenoid, gene expression, and antioxidant analyses.

### 4.8. Carotenoid Extraction and Quantification

Total carotenoid content was determined using a Plant Carotenoids Content Assay Kit (AKPL004C; Beijing Boxbio Science & Technology Co., Ltd., Beijing, China) according to the manufacturer’s instructions. Briefly, pepper fruit tissues were extracted under dark conditions, and absorbance values were recorded at 470, 646, and 663 nm for carotenoid calculation based on standard equations. To provide chromatographic conditions for carotenoid analysis, high-performance liquid chromatography (HPLC) was performed using a reversed-phase C18 column with solvent A (acetonitrile/water, 9:1, *v*/*v*) and solvent B (ethyl acetate). Gradient elution was conducted as follows: 0–25 min, solvent A from 100% to 0%; 25–35 min, solvent B from 0% to 100%; and 35–45 min, solvent A at 100%. The flow rate was 1.0 mL·min^−1^, the column temperature was maintained at 25 °C, the injection volume was 20 μL, and detection was carried out at 472 nm.

### 4.9. Antioxidant Activity Assays

We assessed antioxidant capacity using ABTS^•+^ radical-scavenging and APX activity in a single workflow. ABTS^•+^ was generated from 7.0 mM ABTS + 2.45 mM K_2_S_2_O_8_ (12–16 h, dark, RT) and diluted with phosphate buffer to A_734_ = 0.70 ± 0.02 (1 cm cuvette); plant extracts prepared in phosphate buffer (no organic solvent) were mixed with the working solution and A_734_ was read after 6 min (RT), with Trolox calibration to report TEAC (μMTrolox eq g^−1^ FW). For APX, crude extracts were prepared on ice in 50 mM potassium phosphate (pH 7.0), 1 mM EDTA, 1 mM ascorbate; reactions (1 cm cuvette, RT) contained 50 mM buffer, 0.5 mM ascorbate, 0.25 mM H_2_O_2_, and rates were computed from the decrease in A_290_ using ε_290_ = 2.8 mM^−1^ cm^−1^, expressed as U mg^−1^ protein (Bradford). Data are mean ± SE from *n* = 3 biological × 3 technical replicates.

### 4.10. Yeast Two-Hybrid Screening for CaLCYB1 Interactors

A yeast two-hybrid (Y2H) screening was conducted to identify potential interacting proteins of CaLCYB1. The full-length coding sequence (CDS) of *CaLCYB1* was cloned into the pGBKT7 to generate the GAL4 BD bait. The bait was co-transformed with a pGADT7 prey library or candidate AD constructs into *Saccharomyces cerevisiae* Y2HGold. The yeast transformants were then plated onto selective media, including SD/−Trp/−Leufor initial selection, SD/−Trp/−Leu/−His/−Ade for protein–protein interaction detection, and SD/−Trp/−Leu/−His/−Ade supplemented with X-α-gal for blue/white screening. The plates were incubated at 30 °C for 3–5 days to allow for growth and interaction. Protein–protein interactions were determined by monitoring the growth of co-transformed yeast cells and assessing the β-galactosidase activity using X-α-gal, where positive interactions result in blue colonies. The primers used for vector construction are listed in Table S4.

### 4.11. Subcellular Localization

To determine the subcellular localization of *CaLCYB1*, the full-length CDS without the stop codon was amplified and cloned into a plant expression vector under the control of the CaMV 35S promoter to generate the 35S::CaLCYB1-GFP fusion construct. The empty 35S::GFP vector was used as a control. The recombinant plasmids were introduced into Agrobacterium tumefaciens strain GV3101. Agrobacterium cultures were grown on LB medium supplemented with 25 μg/mL rifampicin and 25 μg/mL kanamycin at 28 °C for 24 h. Positive single colonies were selected and cultured overnight in liquid LB medium. The bacterial cells were then collected and resuspended in infiltration buffer containing 100 mM MES, 100 mM MgCl_2_, and 200 μM acetosyringone (pH 5.6). The cell suspensions were adjusted to an OD600 of 0.8–1.0 and infiltrated into the abaxial side of Nicotiana benthamiana leaves using a needleless syringe. After incubation in the dark for 48 h, GFP fluorescence was observed using a laser scanning confocal microscope (Leica, Wetzlar, Germany). The primers used for vector construction are listed in Table S4.

### 4.12. Statistical Analysis

All data are presented as the mean ± standard deviation (SD) of three independent replicates. Statistical significance was determined using one-way analysis of variance (ANOVA) followed by Bonferroni’s post hoc test for multiple comparisons in Prism 8.0 (GraphPad Software, Boston, MA, USA). The assumptions of normality and homogeneity of variance were checked prior to ANOVA using GraphPad Prism 8.0. Pearson correlation analysis and linear regression were performed in R (version 4.3.3) using the base stats package. Differences with * *p* < 0.05, ** *p* < 0.01, and *** *p* < 0.001 were deemed statistically significant.

## 5. Conclusions

In summary, this study provides a genome-wide identification of the LCY gene family in pepper and functional characterization of *CaLCYB1*, establishing it as a positive regulator of carotenoid accumulation and antioxidant capacity. Using gain- and loss-of-function approaches, we demonstrate that *CaLCYB1* positively regulates carotenoid accumulation and antioxidant capacity: transient overexpression significantly increases β-carotene and total carotenoid contents and enhances ABTS radical scavenging activity and APX enzyme activity, whereas virus-induced silencing leads to clear reductions in these parameters. These findings support a role for *CaLCYB1* in promoting the β,β-branch of carotenoid biosynthesis and contributing to cellular redox homeostasis. The identification of putative interacting proteins, including ubiquitin and RNA-binding proteins, offers initial insights into possible post-translational regulatory mechanisms. Notably, *CaLCYB1* predominantly promoted the accumulation of downstream carotenoids but exerted little effect on the expression of upstream pathway genes. This expression pattern may facilitate the adaptation of carotenoid metabolism in pepper under stress conditions. Future work employing stable genetic transformation and time-resolved multi-omics analyses will be important to further elucidate the regulatory networks involving *CaLCYB1* and other LCY family members, ultimately supporting efforts to enhance carotenoid-associated protective traits in pepper.

## Data Availability

The data that support the findings of this study are available in this article.

## Supplementary Materials

The following supporting information can be downloaded at: https://www.mdpi.com/article/10.3390/plants15091283/s1. Table S1: Properties and localization of CaLCY proteins; Table S2: Sequence data of CaLCY family genes in *Capsicum annuum*; Table S3: Primer sequence for RT-qPCR; Table S4: The special primers for overexpression, gene silencing, yeast two-hybrid interactors, and subcellular localization of *CaLCYB1*.
